# Mechanical Pulmonary Thrombectomy: A Case Report on a Life-Saving Intervention in Acute Pulmonary Embolism

**DOI:** 10.7759/cureus.72741

**Published:** 2024-10-30

**Authors:** Nyvedya Narendra, Olanrewaju Olaitan, Shiju Mathew, Rahul Chivate

**Affiliations:** 1 Department of Medicine, Worcestershire Acute Hospital NHS Trust, Worcetser, GBR; 2 Department of Interventional Radiology, Worcestshire Acute Hospital NHS Trust, Worcester, GBR; 3 Intensive Care Unit, Worcestshire Acute Hospital NHS Trust, Worcester, GBR; 4 Department of Interventional Radiology, Worcestershire Acute Hospital NHS Trust, Worcester, GBR

**Keywords:** covid 19, hyper coagulable state, interventional radiology (ir), mechanical pulmonary thrombectomy, saddle pulmonary embolism

## Abstract

A 69-year-old male presented to the emergency department with sudden shortness of breath, three weeks after recovering from a COVID-19 infection. Despite having no significant prior medical history, the patient rapidly deteriorated, suffering a cardiac arrest. He was resuscitated and diagnosed with a massive saddle pulmonary embolism, confirmed via echocardiogram and computed tomography pulmonary angiography (CTPA). After systemic thrombolysis with alteplase failed to stabilize his condition, mechanical thrombectomy was performed. Catheter-guided aspiration of the thrombus led to substantial hemodynamic improvement. The patient recovered and was discharged four days later. This case highlights the importance of rapid intervention in managing massive pulmonary embolism and the efficacy of percutaneous thrombectomy in cases of hemodynamic instability despite thrombolysis.

## Introduction

A pulmonary embolism (PE) occurs when a pulmonary artery becomes blocked, typically due to an embolus that has travelled from deep veins in the legs or pelvis to the lungs. Pulmonary embolism often leads to symptoms such as shortness of breath, chest pain, and coughing, although the severity of symptoms can vary widely from being completely asymptomatic to causing cardiovascular collapse or even death [1].

Thrombotic complications are a serious concern in COVID-19 due to their association with poor outcomes [2]. The characteristic features of COVID-19-associated coagulopathy include enhanced thrombin production, elevated platelet counts during the initial stages followed by low platelet counts in severe late stages, reduced fibrinolytic activity, and elevated D-dimer levels [3].

Early diagnosis is crucial for managing pulmonary embolism (PE), as the time between symptom onset and death is often brief. In cases of massive PE 50% of patients die within 30 minutes, 70% within 1 hour, and over 85% within 6 hours of symptom onset. PE is often not detected until an autopsy is performed [4].

## Case presentation

A 69-year-old male attended our emergency department, presenting with a sudden onset of shortness of breath. The patient had a history of COVID-19 infection three weeks prior but had no other significant medical history, such as hypertension, diabetes mellitus, hyperlipidaemia, stroke, heart disease, or autoimmune disorders. The patient was not on any regular medications.

Upon arrival, the patient was conscious and talking, with a patent airway. Physical examination revealed cyanosis and the use of accessory muscles for breathing. Auscultation revealed bilateral air entry with no wheezes or crackles. Despite these findings, the patient’s condition rapidly deteriorated.

Within 30 minutes in the emergency department, the patient suffered cardiac arrest. He was resuscitated, intubated, and transferred to the intensive care unit. An adrenaline infusion was initiated at 100 ml/hr. Additional pharmacologic support included 8.4% sodium bicarbonate 100 ml and 20 mg ketamine, followed by a repeated administration of sodium bicarbonate.

**Figure 1:**
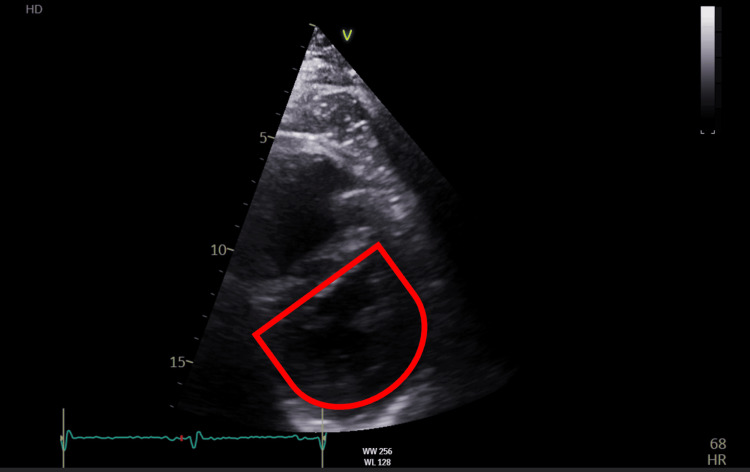
Echocardiogram shows diastolic interventricular septal flattening a sign of right ventricular volume overload - 'D' Shape

**Figure 2:**
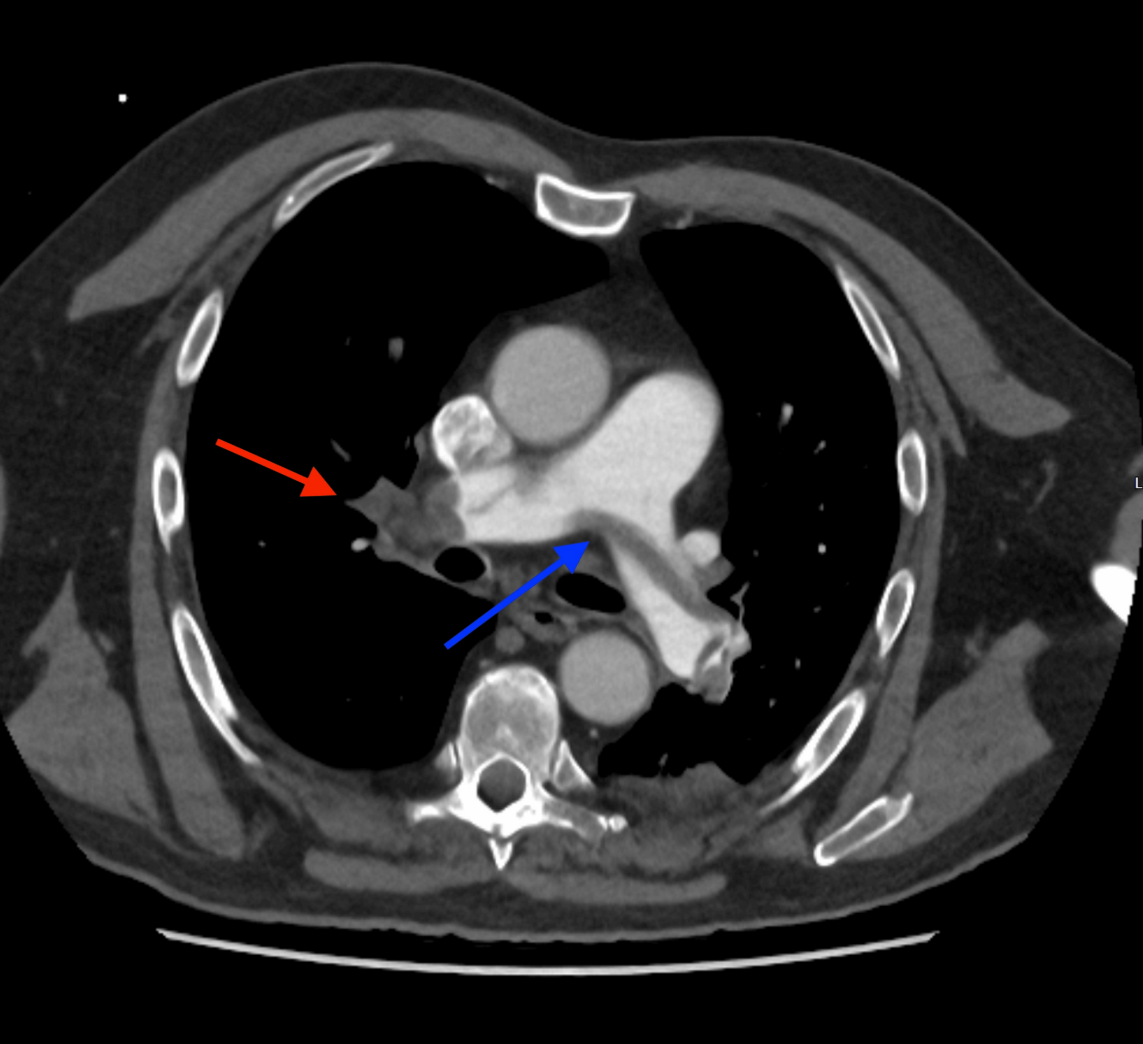
Axial CT pulmonary angiogram shows (blue arrow) saddle thrombus across main pulmonary artery (measuring 3.5 cm) and (red arrow) occlusive thrombus in the right main pulmonary artery.

**Figure 3:**
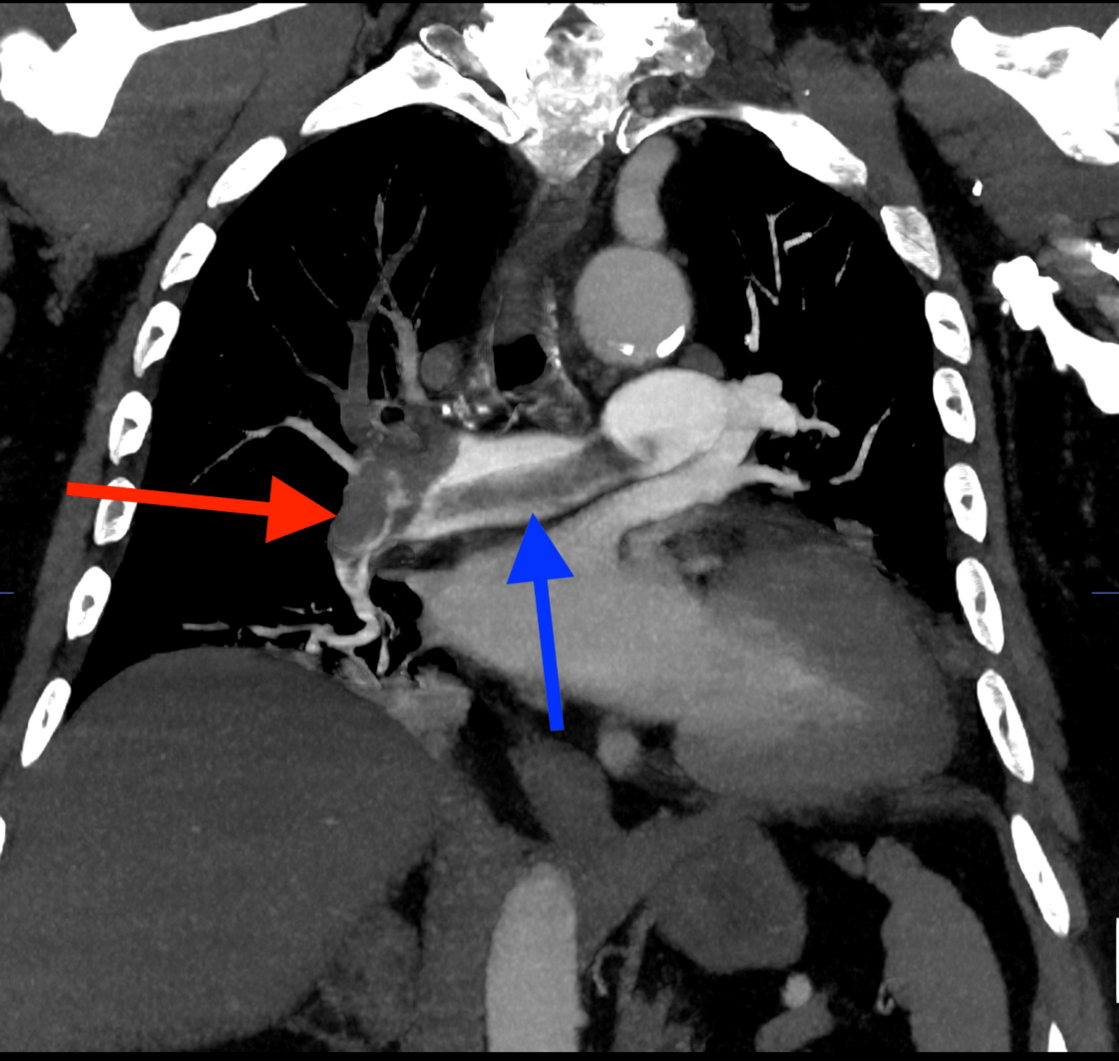
Coronal reconstruction of CT pulmonary angiogram shows (blue arrow) saddle thrombus and (red arrow) thrombus in the right pulmonary artery extending into lobar branches.

A bedside echocardiogram performed in the Accident & Emergency (A&E) department showed evidence of right heart strain as shown in Figure 1. A subsequent computed tomography pulmonary angiography (CTPA) confirmed the diagnosis of a large-volume saddle pulmonary thrombus occluding the right main artery and extending into the lobar branches as shown in Figure 2 and Figure 3. On the left, it was predominantly extending into the left lower lobe artery.

Given the diagnosis of a massive pulmonary embolism, the patient was started on systemic thrombolysis with alteplase. Despite this, the patient’s hemodynamic continued to deteriorate over the next four hours.

Due to the persistent hemodynamic instability, a decision was made to proceed with bilateral pulmonary artery mechanical thrombectomy. Ultrasound-guided access was obtained via the left common femoral vein and no thrombus was visualized in the inferior vena cava or iliac veins.

**Figure 4:**
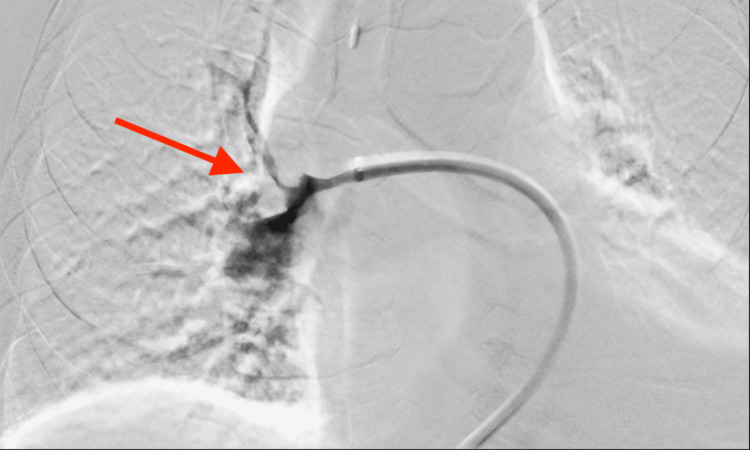
Catheter pulmonary angiogram through Lightning Cat 12, Penumbra shows filling defect in the right pulmonary artery extending into the lobar branches.

Pulmonary artery cannulation was performed and catheter angiography confirmed the findings of CT images as shown in Figure 4.

**Figure 5:**
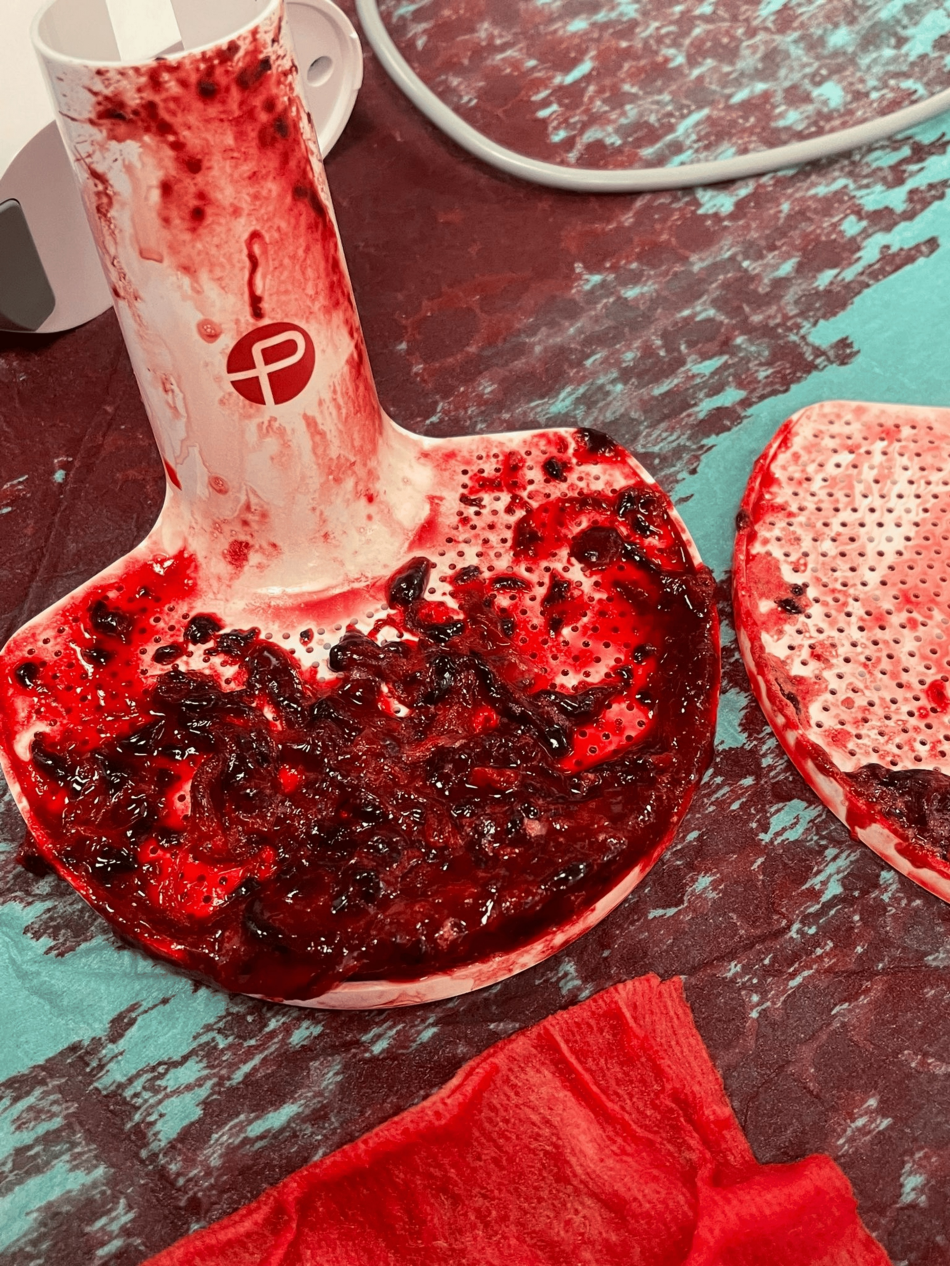
Excavated emboli from pulmonary arteries

**Figure 6:**
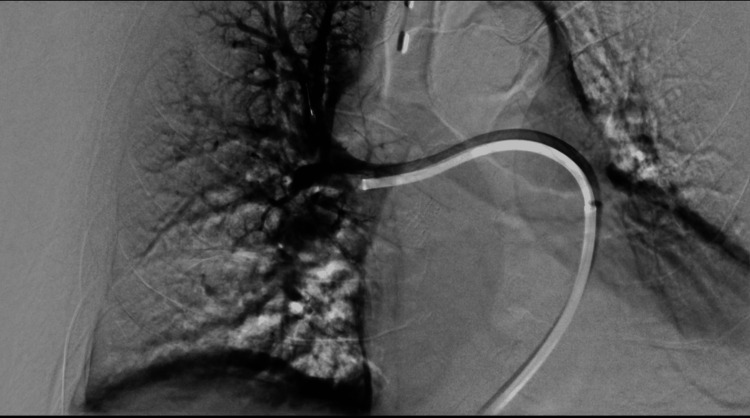
Post thrombus aspiration angiogram shows opacification of right pulmonary artery and its branches.

The patient’s oxygen saturation was 79% on fraction of inspired oxygen (FiO2) 100% (ventilator support) and the pulmonary arterial pressure was 30 mmHg. A Penumbra aspiration device with a Lightning Cat 12 catheter was used for the aspiration of the thrombus as shown in Figure 5. Aspiration of clots resulted in an improvement in oxygen saturation to 94% at room air and a decrease in pulmonary arterial pressure to 23 mmHg. However, the patient experienced a subsequent drop in oxygen saturation due to re-clotting. At this time, the activated clotting time (ACT) was 172 seconds prompting administration of heparin to maintain the activated clotting time (ACT) above 200 seconds. Re-aspiration was performed, achieving a final oxygen saturation of 97% at room air and a pulmonary arterial pressure of 16 mmHg. The angiographic image demonstrated optimal clearance of clots from the bilateral pulmonary arteries as shown in Figure 6.

The patient improved gradually and was taken off vasopressor support. He was extubated the next day and discharged home after 4 days. The comparison between the observations pre and post procedure are mentioned in Table 1.

**Table 1:** Observations Pre and Post Procedure SBP: Systolic blood pressure; DBP: Diastolic blood pressure

Observations	On Arrival	On Discharge	Reference Range
Blood Pressure (mm of Hg)	177/112	142/78	SBP:100-140 DBP: 60-90
Heart Rate (beats per minute)	133	92	60-100
Respiratory Rate (cycles per minute)	48	19	12-20
Saturation	52% at 15 litres of 02	97% at room air	100% at room air
Cardiac Troponin-I (pg/ml)	296	1040	>50
Lactate (mmol/l)	8.8	0.7	0.5-1

## Discussion

The first reported case of pulmonary embolism (PE) caused by COVID-19, along with acute heart failure occurred on 16th March 2020. The incidence of thrombotic events in COVID-19 patients has been noted to reach up to 79% [2]. Endothelial cell damage caused by SARS-CoV-2 infection leads to impaired physiological anticoagulant function promoting a more procoagulant and thrombotic state. The heightened coagulation observed in COVID-19 likely results from the activation of clotting pathways, reduced fibrinolysis, and immune responses that initiate immunothrombosis [5].

In our patient, symptoms appeared three weeks after recovering from a COVID-19 infection. Notably, he had no prior history of significant medical conditions nor was he on any regular medications. His recent COVID-19 infection and the nature of his symptoms strongly suggested that he developed a COVID-19-related thrombotic event resulting in a pulmonary embolism.

A patient with pulmonary embolism should be assessed for outpatient management if they are low risk. If suitable, they should receive treatment in an outpatient setting with appropriate follow-up. The guidelines recommend using validated clinical risk scores like Pulmonary Embolism Severity Index (PESI) and simplified PESI (sPESI). Those with low scores may be managed as outpatients provided they do not have exclusion criteria like hemodynamic instability, active bleeding, or severe pain [6]. A massive PE is characterized by persistent low blood pressure or shock, indicating a high-risk situation. In contrast, a sub-massive PE involves dysfunction of the right ventricle or injury to the heart muscle, but without causing significant blood pressure changes or shock, placing it in the intermediate-risk category [1].

The British Thoracic Society (BTS) guidelines on the initial outpatient management of pulmonary embolism (PE) include critical tools for risk stratification, namely the Pulmonary Embolism Severity Index (PESI), simplified PESI (sPESI), and the Geneva score. These clinical scores are pivotal in determining the risk level of patients with pulmonary embolism, guiding whether they can be managed safely as outpatients or require inpatient care [6].

The PESI score is derived from a large cohort study and is used to predict 30-day mortality in patients with pulmonary embolism. It considers factors like age, sex, cancer, heart failure, chronic lung disease, pulse rate, systolic blood pressure, respiratory rate, temperature, mental status, and arterial oxygen saturation. Patients are classified into five risk classes (I to V) with corresponding scores:

Class I: Very Low Risk (≤65 points)

Class II: Low Risk (66-85 points)

Class III: Intermediate Risk (86-105 points)

Class IV: High Risk (106-125 points)

Class V: Very High Risk (≥126 points)

PESI has been extensively validated and typically identifies around 50% of patients as low-risk with very low associated mortality. The sPESI simplifies the original PESI by focusing on six key variables: age > 80 years, history of cancer, chronic cardiopulmonary disease, pulse ≥ 110 bpm, systolic blood pressure < 100 mm Hg, and arterial oxygen saturation < 90%. A score of 0 is considered low risk, while a score of ≥1 is high risk. The sPESI has been shown to be non-inferior to the original PESI in predicting 30-day mortality with a similarly low incidence of major bleeding and recurrent VTE in low-risk patients [6]. Based on the Pulmonary Embolism Severity Index (PESI) score, our patient falls into the "Class V: Very High Risk" category. This classification highlights the critical need for inpatient care due to the elevated risk of mortality.

The Geneva score, which includes clinical and subjective criteria predicts outcomes in PE patients, with an emphasis on factors such as cancer and heart failure. However, the Geneva score generally categorizes more patients as low-risk compared to PESI, though it has been found to be less effective at identifying those at the lowest risk of mortality. These scores aid clinicians in deciding the appropriate management pathway for PE patients. Low-risk patients, as identified by these tools, are often suitable for outpatient management, leading to reduced hospital stays and high patient satisfaction. Patients managed as outpatients are still closely monitored to ensure that they do not develop complications, with specific follow-up protocols based on their risk profile [6].

In the case of acute pulmonary embolism large community studies indicate that the overall annual incidence of pulmonary embolism (PE) is 60-70 cases per 100,000 people. About half of these patients develop venous thromboembolism (VTE) while in the hospital or in long-term care, with the remaining cases evenly split between idiopathic cases and those with known risk factors. In various studies, both representative and less so, in-hospital mortality rates for PE ranged from 6% to 15%. In the most comprehensive and representative cohort study, of the 814 patients who initially survived, 7% died within 1 week, 13% within 1 month, and 18% by 3 months. These studies consistently found that a significant proportion of early deaths were directly attributable to PE despite standard treatment. The risk of venous thromboembolism (VTE) increases exponentially with age, though it's unclear if age alone is an independent risk factor. The widespread use of prophylaxis in orthopaedic and general surgery has significantly reduced the incidence of post-operative VTE. In addition, air and road travel, particularly for long journeys is associated with a 2-4 times increased risk of VTE. Thrombophilia is identified in 25-50% of VTE cases especially when combined with other risk factors. The factor V Leiden mutation is present in 5% of the general population but in 20% of those presenting with thrombosis, increasing VTE risk by 3-5 times. This risk can rise to 35 times when combined with oestrogen therapy. After a first VTE episode, there is a 7-12% chance of detecting previously unrecognized cancer within 6-12 months, particularly in cases of idiopathic VTE. The 1-year survival rate for patients with occult cancer discovered after a VTE episode is only 12%. Thrombophilia testing is recommended for patients under 50 with recurrent PE or a strong family history of VTE. Cancer investigation is only indicated in idiopathic VTE if cancer is clinically suspected or indicated by routine tests [7].

Massive pulmonary embolism (PE) represents less than 10% of all acute PE cases and is a medical emergency with a high mortality rate. The primary treatment for PE is systemic anticoagulation. In cases of massive or sub-massive PE, systemic thrombolysis may be administered, and in rare instances, open surgical embolectomy is performed. Catheter-directed therapies such as catheter-directed thrombolysis (CDT) and percutaneous thrombectomy, may also be utilized. Percutaneous thrombectomy is typically used for patients with massive PE who cannot undergo thrombolysis due to contraindications, when thrombolysis has failed, or when surgery is not an option [1].

In the case of our patient, who was diagnosed with a massive pulmonary embolism (PE), initial treatment followed standard protocols with systemic thrombolysis using alteplase. This aligns with typical management for massive PE, where systemic thrombolysis is considered in cases with significant hemodynamic compromise. However, despite receiving thrombolysis, the patient's condition continued to worsen, indicating that the intervention was not sufficient to stabilize his hemodynamics. Given the patient’s persistent instability, a decision was made to perform a bilateral pulmonary artery mechanical thrombectomy. This approach is consistent with literature recommendations for massive PE patients who are unresponsive to thrombolysis and remain hemodynamically unstable. Thus, offering a life-saving intervention when surgery is not viable.

## Conclusions

This case highlights the successful diagnosis and management of a massive pulmonary embolism (PE) with a history of recent COVID-19 infection. Despite initial systemic thrombolysis with alteplase, the patient’s hemodynamic status deteriorated, necessitating an emergent bilateral pulmonary artery mechanical thrombectomy. The patient's recovery was uneventful, and he was successfully extubated the next day and discharged in stable condition after four days. This case underscores the importance of timely intervention in managing high-risk PE cases and highlights the efficacy of mechanical thrombectomy in restoring pulmonary function and reducing mortality. Given the statistical correlation between COVID-19 and pulmonary embolism (PE), the PESI and Geneva scoring systems can be considered to include COVID-19.

In conclusion, mechanical pulmonary thrombectomy is a lifesaving procedure. It is essential to maintain the activated clotting time (ACT) level to an optimum level during and after the procedure to prevent recurrent thrombosis.
